# Supplementary material for: Online randomised trials with children: A scoping review protocol
Source: HRB Open Res. 2022 Jun 20;5:46. [Version 1] doi: 10.12688/hrbopenres.13566.1 (PMC10238819; doi:10.12688/hrbopenres.13566.1)
Supplement: Peer review history

# Peer review: Reviewer response for version 1

**DOI:** 10.21956/hrbopenres.14812.r33773

**Published:** 2023-06-02

**Authors:** Niina Kolehmainen

**Affiliations:** 1 Newcastle University, Newcastle upon Tyne, UK

Thank you for the opportunity to review this very timely and interesting protocol paper.

 ABSTRACT:

 Methods should say the key review question, and the key search facets and main inclusion criteria. If short of words to do this, there is scope to shorten the introduction and take out obvious statements (e.g. “Once identified”) to ensure all key aspects of methods are covered.

 INTRO:

 This is well articulated and clearly written, covering the key points and outlining the justification. It provides a helpful summary of many key issues, and directs reader to relevant references.

 As a small point, the term “hybrid trial” is used in various ways in different trials related literature – unless this is a key term for the paper, it would seem better to leave this out and just provide a slightly fuller statement of what the authors mean, e.g. “…publications from trials that combine in persona and online approaches…” (it is currently not clear what the authors mean by this term).

 METHODS:

 These are clearly written and cover all the expected points in sufficient detail.

 The only addition is that the inclusion list should include protocols (as this is where the methods are most likely reported in the detail that the authors need, to answer the question).

 DISCUSSION:

 I appreciate this being brief and to the point.

 Overall: this paper does what a good protocol paper should do, i.e. it reports in advance the methods intended to be deployed, and does so clearly and in detail that would make most of the review replicable. It is very likely that the authors will face further decisions as they progress the review (it almost always happens in these type of reviews) but that is fine at this stage and these can be reported down the line, alongside the results.

Is the study design appropriate for the research question?

Yes

Is the rationale for, and objectives of, the study clearly described?

Yes

Are sufficient details of the methods provided to allow replication by others?

Yes

Are the datasets clearly presented in a useable and accessible format?

Yes

Reviewer Expertise:

Child health, complex intervention methods, evidence synthesis methods

I confirm that I have read this submission and believe that I have an appropriate level of expertise to confirm that it is of an acceptable scientific standard.

# Peer review: Reviewer response for version 1

**DOI:** 10.21956/hrbopenres.14812.r32677

**Published:** 2022-08-16

**Authors:** Peter Knapp

**Affiliations:** 1 Department of Health Sciences and the Hull York Medical School, University of York, York, UK

The submitted protocol is clearly written and well-structured and referenced. The review is timely and well-justified. 

 The aim of the review is to understand “How have online, randomised trials been conducted with children?”. My only concern is that the published reports (that will be included) might not include online trials that were planned but not enacted, or were started but not completed. I have no idea how common these outcomes occur (or whether the rate varies by comparison with non-online trials or online trials with adults) but it's an important outcome. Could the scoping review be complemented by a survey of those involved in children's trials (a bit messy but could give useful indications)?

Is the study design appropriate for the research question?

Yes

Is the rationale for, and objectives of, the study clearly described?

Yes

Are sufficient details of the methods provided to allow replication by others?

Yes

Are the datasets clearly presented in a useable and accessible format?

Not applicable

Reviewer Expertise:

Information for patients, including information about trials.

I confirm that I have read this submission and believe that I have an appropriate level of expertise to confirm that it is of an acceptable scientific standard.
